# Making connections

**DOI:** 10.7554/eLife.05504

**Published:** 2014-11-19

**Authors:** Justin W Kenney, Paul W Frankland

**Affiliations:** 1 **Justin W Kenney** is in the Neurosciences and Mental Health Program, Hospital for Sick Children, Toronto, Canada; 2 **Paul W Frankland** is in the Neurosciences and Mental Health Program, Hospital for Sick Children, Toronto, Canada; Department of Psychology, Institute of Medical Science and Department of Physiology, University of Toronto, Toronto, Canadapaul.frankland@sickkids.ca

**Keywords:** synaptogenesis, semaphorin, hippocampus, dentate granule cells, plexin, 3D electron microscopy, mouse

## Abstract

Deleting a gene called *Sema5A*, which has been linked to autism in humans, causes neurons to form more connections in mice, and also alters how these mutant mice interact with other mice.

**Related research article** Duan Y, Wang SH, Song J, Mironova Y, Ming GL, Kolodkin AL, Giger RJ. 2014. Semaphorin 5A inhibits synaptogenesis in early postnatal- and adult-born hippocampal dentate granule cells *eLife* **3**:e04390. doi: 10.7554/eLife.04390.

**Image** Genes linked to autism and related disorders control the density of synapses in a specific region of the brain

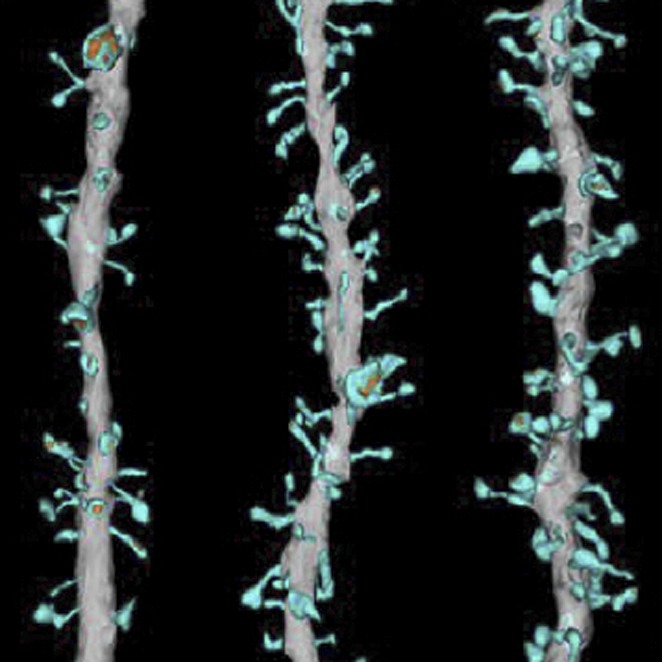

Autism spectrum disorders—such as Asperger syndrome and childhood autism—have long been known to have a significant genetic component (Ronald and Hoekstra, 2011). However, the biological basis of these disorders has remained largely elusive. Hundreds of genes have been linked to autism spectrum disorders (or ASDs), but most only contribute a small increase in risk (Devlin and Scherer, 2012).

It has been proposed that ASDs are caused by the abnormal development or dysfunction of synapses: these are the connections between neurons that allow chemical signals to be sent from one neuron to another in the brain (Sudhof, 2008). The dysfunction is thought to lead to a change in the relative numbers of excitatory and inhibitory signals received by neurons. Now, in *eLife*, Roman Giger and colleagues at the University of Michigan School of Medicine and the Johns Hopkins University School of Medicine report additional evidence for a link between ASDs and dysfunctional synapses (Duan et al., 2014).

Previous studies have shown that small genetic differences near a human gene called *SEMA5A* confer an increased risk for ASDs. It has also been shown that *SEMA5A* expression is reduced in the brains of people with ASDs (Weiss et al., 2009). The *SEMA5A* gene encodes a transmembrane protein called Semaphorin 5A that is important for guiding the branches of neurons during development (Kantor et al., 2004). Consistent with a prominent role of synapses in ASDs, Giger and colleagues—who include Yuntoa Duan and Shih-Hsiu Wang as joint first authors—report that deleting the *Sema5A* gene from mice results in an increase in the density of synapses and in increased transmission of excitatory signals across synapses (Figure 1). The increase in synaptic density appears in granule cells in a region of the brain called the dentate gyrus, which is found in the hippocampus.

**Figure 1.**
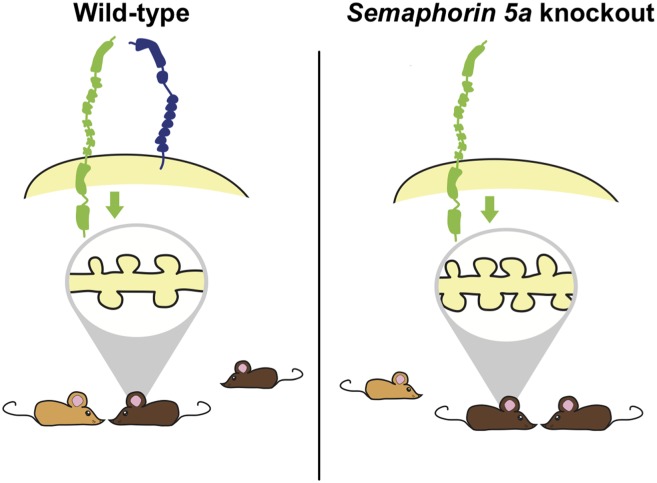
Deleting the gene for Semaphorin 5A in mice increases the density of synapses and changes how mutant mice interact with other mice. Duan, Wang et al. report that semaphorin 5A (blue) inhibits the formation of synapses (shown inside the grey oval; left) in granule cells in the dentate gyrus of the hippocampus by interacting with a protein called plexin-A2 (green). This occurs in granule cells that developed in the embryo and have matured, and also in granule cells that formed during adulthood. In the absence of Semaphorin 5A, the density of synapses increases (right). Wild-type mice (bottom left) prefer to interact with an unfamiliar mouse (light brown) over a familiar mouse (dark brown). In contrast, *Sema5A* knockout mice (bottom right) show greater preference for a familiar mouse, which mimics the social anxiety seen in autism spectrum disorders.

At the behavioural level, ASDs are characterised by a combination of abnormal social interactions, difficulties with communication and repetitive behaviours. Alterations in ASD genes related to synaptic function typically result in deficits in social interactions in mice (Ey et al., 2011). Consistent with this previous work, Duan, Wang et al. report that mice in which the *Sema5A* gene has been ‘knocked-out’ perform poorly in a social preference task, but show normal levels of performance in several other learning tasks or behaviours. Such specific deficits suggest that different behaviours observed in ASDs may be underpinned by distinct biological processes. However, it is not clear how an increase in the synaptic density in granule cells of the dentate gyrus leads to deficits in social interactions.

The traditional view of developmental disorders of the brain and nervous system holds that there is a limited time window during development in which treatment may be beneficial. From this perspective, treatment during adulthood would be of limited value. However, several studies over the last decade using mouse models of autism and related disorders have challenged this perspective. For several of these mouse models, treatment with drugs enable adult mice to overcome both their cognitive and social interaction deficits (Ehninger et al., 2008; Castren et al., 2012). Duan, Wang et al. find that treating neurons grown in the laboratory with a fragment of the Sema5A protein fused to another molecule for only two hours decreases the density of synapses. This suggests that the structural abnormalities in neurons that are thought to underlie ASDs are highly plastic and amenable to manipulation on short time scales. However, it remains to be seen whether such rapid effects are also observed in living mice and whether they are capable of mitigating the behavioural deficits.

A key goal in unveiling the genetic underpinnings of any disorder is to provide a basis for the rational design of therapeutics. Towards this end, Duan, Wang et al. identified a protein called plexin-A2 (or PlxnA2) as the receptor that mediates the effects of Semaphorin 5A on synaptic density. They find that Semaphorin 5A binds to plexin-A2 to restrict the growth and density of synapses in individual neurons. Like *Sema5A* knockout mice*, PlxnA2* knockout mice have more synapses on the granule cells in the dentate gyrus. Furthermore, synapse density was not affected by over-production of Semaphorin 5A in neurons with the *PlxnA2* gene deleted when grown in vitro. This suggests that the plexin-A2 protein may be a useful target to explore with respect to developing novel therapeutics for ASDs and other disorders in which there is an increase in synaptic density.

Although the biological and genetic bases for ASDs are being slowly unravelled, numerous questions still remain. Of the most perplexing is how changes in synapse number or function lead to specific deficits, such as difficulties with social interaction, and do not have more general behavioural effects. Of potentially greater consequence is whether or not treatment, pharmacological or otherwise, will be beneficial in older children and adults. Our search for the physiological basis of ASDs promises not only to eventually yield treatments for these disorders, but also to deepen our understanding of normal workings of synapses.
